# First Report of *Meloidogyne Morocciensis* Infecting Hops (*Humulus Lupulus*)

**DOI:** 10.2478/jofnem-2022-0005

**Published:** 2022-04-11

**Authors:** Eduardo France Oza, José Olívio Lopes Vieira Júnior, Millena Monteiro dos Santos, Mariana Zandomênico Mangeiro, Renata Cunha Pereira, Ricardo Moreira Souza, Antônio Fernando de Souza, Silverior de Paiva Freitas

**Affiliations:** 1 Universidade Estadual Norte Fluminense Darcy Ribeiro, Campos dos Goytacazes, Rio de Janeiro, Brazil; 2 Centro Universitário Norte do Espírito Santo, Universidade Federal do Espírito Santo, São Mateus, Espírito Santo, Brazil; 3 Instituto Federal de Ciência e Tecnologia do Espírito Santo, Santo Teresa, Espírito Santo, Brazil

**Keywords:** diagnosis, first occurrence, nematodes

## Abstract

*Humulus lupulus* (Cannabaceae) is a climbing herbaceous plant with perennial production, intended mainly for the brewing industry. *H. lupulus* is widely cultivated in temperate regions; hop cultivars have shown good adaptation in regions of Brazil. In a hop-growing area in São Mateus, the state of Espírito Santo, leaf wilting and galling of the root system was observed. Soil and root samples were taken to the laboratory and processed, and the nematodes extracted from the *Meloidogyne* genus were identified by morphology, morphometry, and biochemical analysis. According to the results, the species identified in the hop roots was *Meloidogyne morocciensis*. This is the first report of *H. lupulus* as host of *M. morocciensis*.

Hop (*Humulus lupulus* L.) is a climbing herbaceous plant (Fig. 1A) belonging to the Cannabaceae family, native to temperate regions. Flowers of this plant are mainly used in the brewing industry for conferring aroma and flavor to beer, but since it has secondary metabolites with significant biological activities (Astray *et al*., 2020), the plant is also applied in medicine and can be used in the treatment of diseases, such as insomnia, stress, and anxiety (Kyrou *et al*., 2017). Although Brazil is a country with a tropical climate, *H. lupulus* has adapted in regions with a mild climate and shows good plant development (Durello *et al*., 2019).

**Figure 1:**
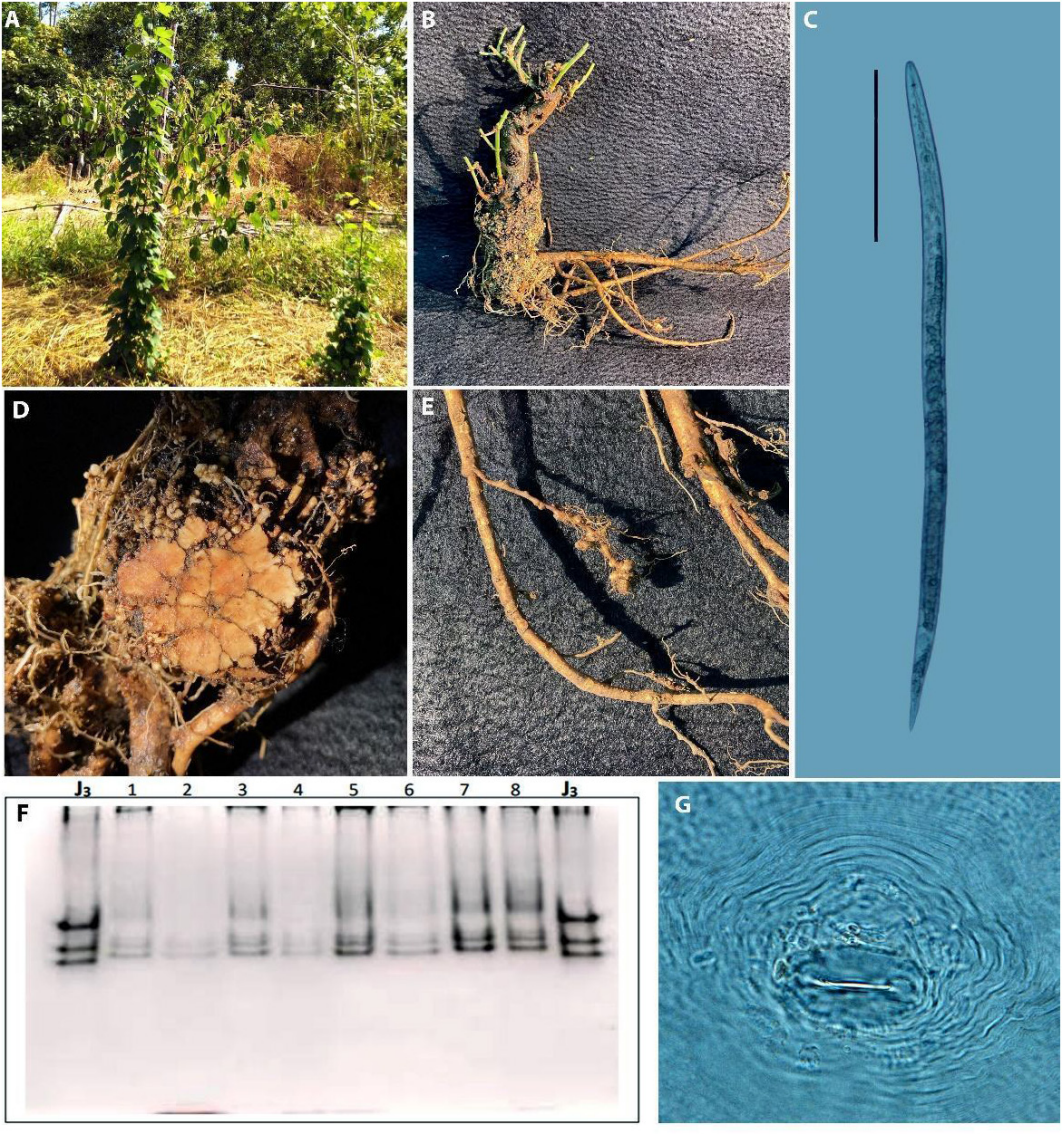
(A) Hop plants with leaf wilt symptoms; (B) hop root with symptoms of swelling and galls on roots; (C) juvenile (J2) of *Meloidogyne morocciensis*, reference scale: 50 μm; (D) cross-section of infected roots with galls; (E) root galls formed by *M. morocciensis*; (F) PAGE analysis of esterase isoenzyme phenotype, with one female in each cavity (J3 = *M. javanica* and 1–8: females extracted from hop roots); (G) perineal region of a female of *M. morocciensis*.

Seedlings of *H. lupulus* of the Cascade variety obtained through vegetative propagation of branches of a healthy hop plant were planted in the municipality of São Mateus, in the northern region of the state of Espírito Santo (18° 40′ 25″ S, 40° 51′ 23″ W; altitude of 77 m) in July 2019. Eight months after transplanting, the plants showed symptoms of leaf yellowing, reduced development evolving to leaf wilting, and senescence of the aerial part. After observation of the presence of root galls (Fig. 1B,D,E), soil and root samples were collected from the rhizosphere of five hop plants. Four soil subsamples were collected from each plant and homogenized in plastic bags to form five composite soil samples (*n* = 5). To sample the roots, five plants were collected from the soil, taking care to keep the root system intact, and placed in a plastic bag. The soil and root samples were taken to the Nematology Laboratory of the Universidade Estadual do Norte Fluminense Darcy Ribeiro, the Nematology Laboratory of the Universidade Federal de Lavras, and the Phytopathological Analysis Laboratory of the Universidade Federal do Espírito Santo for nematological analyses.

Nematodes were extracted from soil using the method of Jenkins (1964) and from roots using the method of Coleen and D’Herde (1972). For the identification of *Meloidogyne* species, the morphology of female perineal patterns was used (*n* = 10), morphometry of juveniles (*n* = 20), and by esterase phenotypes obtained by electrophoresis gel (*n* = 8). Eggs and juveniles were counted on Peter’s slides to quantify the population found in the samples.

According to the results of the analyses, it was possible to identify the species as *Meloidogyne mococciensis* Rammah and Hirschmann, 1990 and as a parasite of the hop plants. The estimated population of eggs and juveniles was 864/100 cm^3^ in soil and 2,763/g root. The perineal patterns found exhibit oval-to-square shapes, with moderately separated dorsal arches and continuous and separate coarse striations, similar to that found by Machaca-Calsin *et al*. (2021) (Fig. 1G).

The measurements of second-stage (J2) juveniles (*n* = 20) (Fig. 1C) were as follows: *L* = 408.84 ± 3.46 (380.98–434.31) μm; stylet length: 13.61 ± 0.17 (12.48–15.08) μm; dorsal gland opening: 3.67 ± 0.08 (2.99–4.26) μm; tail length: 48.62 ± 0.39 (45.91–51.03) μm; hyaline tail length: 15.96 ± 0.25 (14.73–17.85) μm; a: 25.98 ± 0.42 (23.2–27.85) μm; and c: 7.14 ± 0.18 (6.99–7.91) μm. No males were found for the morphological and morphometric analyses. According to Rammah and Hirschmann (1990), the measurements obtained from the second-stage (J2) juveniles correspond to those of the species *M. morocciensis*. The esterase isoenzyme phenotype was characterized as A3 by PAGE (Fig. 1F) in the esterase identification method (Carneiro *et al*., 2008).

*Meloidogyne morocciensis* is a species less frequently found worldwide when compared to traditional species such as *M. javanica* and *M. incognita*. In Brazil, this nematode has been found in beetroots (Machaca-Calsin *et al*., 2021), peach trees (Silva *et al*., 2020), tomato plants (Barros *et al*., 2018), and soybean (Dalla Nora *et al*., 2020). Based on the results of the species identification analyses, this is the first report of *M. morocciensis* on hop roots. We observed that infected hop plants showed developmental damage, such as reduced growth, chlorosis on leaves, and nodules on roots. The disease could reduce hop production and affect other crops reported to be hosts of economic importance in Brazil, such as soybeans and tomatoes. Thus, we recommend hop growers in Brazil, especially in the northern region of Espírito Santo, to take care and avoid the spread of this nematode to other agricultural crops and to other regions of the country.
